# Ternary Logic Design Based on Novel Tunneling-Drift-Diffusion Field-Effect Transistors

**DOI:** 10.3390/nano15161240

**Published:** 2025-08-13

**Authors:** Bin Lu, Hua Qiang, Dawei Wang, Xiaojing Cui, Jiayu Di, Yuanhao Miao, Zhuofan Wang, Jiangang Yu

**Affiliations:** 1 School of Physics and Information Engineering, Shanxi Normal University, Taiyuan 030031, China; lubinsxnu@sina.cn (B.L.); 17200671507@163.com (H.Q.); wdw0301@163.com (D.W.); 20210084@sxnu.edu.cn (X.C.); dijiayu@sxnu.edu.cn (J.D.); 2 Jiachuang Semiconductor Technology, Co., Ltd., Jinjiang 362200, China; 3 Research and Development Center of Optoelectronic Hybrid IC, Guangdong Greater Bay Area Institute of Integrated Circuit and System, Guangzhou 510535, China; miaoyuanhao@giics.com.cn; 4 School of Information Engineering, Xi’an University, Xi’an 710065, China; wangzhuofan@xawl.edu.cn; 5 State Key Laboratory of Widegap Semiconductor Optoelectronic Materials and Technologies, North University of China, Taiyuan 030051, China

**Keywords:** hybrid conduction mechanism, ternary inverter, combinational ternary logic circuits, sequential ternary logic circuits

## Abstract

In this paper, a novel Tunneling-Drift-Diffusion Field-Effect Transistor (TDDFET) based on the combination of the quantum tunneling and conventional drift-diffusion mechanisms is proposed for the design of ternary logic circuits. The working principle of the TDDFET is analyzed in detail. Then, the device is packaged as a “black box” based on the table lookup method and further embedded into the HSPICE platform using the Verilog-A language. The basic unit circuits, such as the Standard Ternary Inverter (STI), Negative Ternary Inverter (NTI), Positive Ternary Inverter (PTI), Ternary NAND gate (T-NAND), and Ternary NOR gate (T-NOR), are designed. In addition, based on the designed unit circuits, the combinational logic circuits, such as the Ternary Encoder (T-Encoder), Ternary Decoder (T-Decoder), and Ternary Half Adder (T-HA), and the sequential logic circuits, such as the Ternary D-Latch and edge-triggered Ternary D Flip-Flop (T-DFF), are built, which has important significance for the subsequent investigation of ternary logic circuits.

## 1. Introduction

With the rapid development of the Internet of Things (IoT) and artificial intelligence (AI), modern information technology shows increasing demand for improving data storage and processing capabilities [1,2]. Advances in integrated circuit (IC) technology are increasingly focusing on low power consumption and high integration to meet the challenges. In order to improve the performance of the IC, the feature size of transistors is continuously reduced [3,4,5,6]. However, with the transistors scaled to the nano-scale, the short-channel effects lead to increasingly significant static power consumption in the CMOS technology [7]. At present, the reduction in the transistor size has approached the physical limit determined by fundamental laws [8,9]. As the complexity of data and algorithms increases, traditional binary systems are increasingly limited in handling and managing complex computational requirements. In contrast, the multi-valued systems are increasingly considered as strong candidates for replacing the traditional binary systems [10,11].

Three is generally considered the optimal logical cardinal number, considering it is the integer closest to the natural constant, and the ternary logic presents obvious advantages over the binary logic [12]. For example, ternary logic can obtain a higher density of information with the same number of gates or chip area as binary logic, and it requires fewer gates to store the same information. Ternary logic allows more information to be efficiently transmitted over a given set of wires and more data to be compactly stored in a fixed register length [13,14,15,16,17]. As a result, the complexity associated with the chip inter-connectivity and space requirements can be significantly reduced. Specifically, compared to the binary logic, the ternary logic system achieves a 36.9% reduction in chip complexity and greatly improves the utilization of hardware resources [18]. As an extension of the traditional logic system, the ternary logic system provides a new perspective for understanding and dealing with uncertainty. By introducing a third state, i.e., “uncertainty”, it enriches the dimension of logical expression and allows AI systems to deal with ambiguous and incomplete information with greater flexibility [19,20,21].

A variety of novel devices, including the quantum dot gate field-effect transistors (QDGFETs) [22], memristors [13,23], negatively-capacitor field-effect transistors (NCFETs) [24], carbon nanotube field-effect transistors (CNTFETs), and graphene [25] nanoribbon field-effect transistors (GNRFETs) [26,27], have shown potential for building ternary logic circuits. However, these devices usually require additional passive components or multi-valued power supplies when constructing the ternary logic inverter, which increases the chip area and system complexity, and does not have the advantages of the ternary logic itself. In addition, the application of novel low-dimensional materials also leads to significant challenges when integrating these devices into the traditional complementary metal-oxide-semiconductor (CMOS) manufacturing processes, and there are considerable difficulties in scaling these devices to the levels required for very large-scale integrated (VLSI) circuits.

To solve these problems, a novel Tunneling-Drift-Diffusion Field-Effect Transistor (TDDFET) is proposed by combining the carrier tunneling mechanism with the conventional drift-diffusion mechanism, enabling the TDDFET to exhibit a third stable state in addition to the traditional on and off states, making it very suitable for the ternary logic design. The TDDFET that is only based on the traditional silicon material and does not require novel material can maximize the compatibility with the traditional CMOS platform. In addition, compared to the already published design for the ternary inverters, the TDDFET-based ternary inverter requires only two devices and involves no passive component or multi-valued power supply. This is very significant for reducing the chip area and system complexity as well as improving the integration.

## 2. Device Structure and Principle

**Figure 1:**
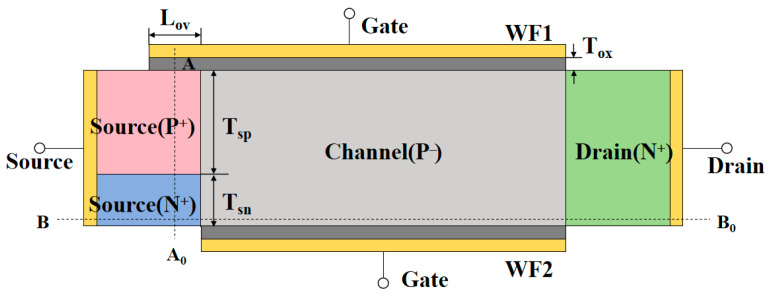
The structure of the proposed n-type TDDFET.

Figure 1 shows the structural diagram of the proposed n-type Si TDDFET (nTDDFET), and the key parameters are presented in Table 1. The source is composed of N+ and P+ doped regions. In addition, the device has two asymmetrical gates at the top and bottom, with the top gate extending to cover a portion of the P+ source, while the bottom gate is aligned with the channel region and covers only the channel body. The P+ source, top gate, channel body, and N+ drain form a p-i-n TFET, while the N+ source, bottom gate, channel body, and N+ drain form an n-i-n MOSFET. The P+ source of the TFET is electrically connected to the N+ source of the MOSFET and shares the channel body and drain region; so, the two devices are electrically connected in parallel. In order to facilitate the subsequent analysis, two cut-lines, AA_0_ and BB_0_, are also marked in Figure 1, where AA_0_, located at the midpoint of the overlap area between the top gate and P+ source, is vertical to the channel direction, and BB_0_, located 3 nm above the bottom surface of the channel body, is parallel to the channel direction.

**Figure 2:**
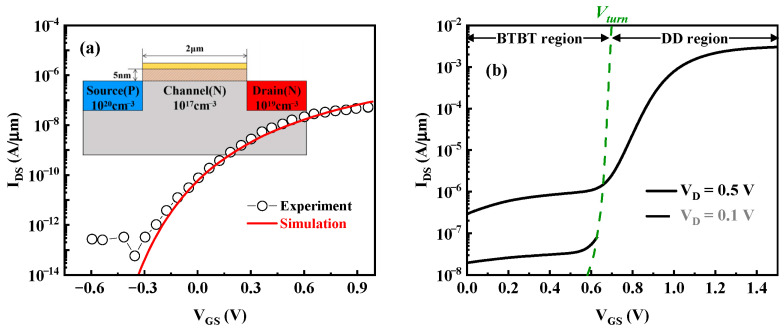
The tunneling model calibrated with the experimental Si-based TFET in [28] (**a**,**b**) the transfer curves of the proposed n-type TDDFET.

In this work, the TCAD Sentaurus is used to analyze the principle and performance of the device. The dynamic non-local BTBT model, which considers the influence of a non-uniform electric field on the tunneling probability, is used to simulate the tunneling current and is calibrated by the same device structure as the experimental TFET reported in [28], as shown in Figure 2a. In addition, the mobility model related to the doping concentration, band gap narrowing, Shockley–Read–Hall recombination model, and high electric field velocity saturation model are also considered.

Figure 2b exhibits the transfer curves of the n-type TDDFET at V_DS_ = 0.1 V and V_DS_ = 0.5 V. These curves are significantly different from those observed for MOSFETs and TFETs. The curves show a significant turn voltage, V_turn_. For V_GS_ < V_turn_, the drain current I_DS_ increases slowly with V_GS_, and once V_GS_ > V_turn_, I_DS_ increases rapidly with V_GS_ and then gradually approaches saturation. It is clear that the law of the transfer curve when V_GS_ is larger than V_turn_ is more similar to that of MOSFETs. The two different current trends before and after V_turn_ indicate two different conduction mechanisms of current.

**Figure 3:**
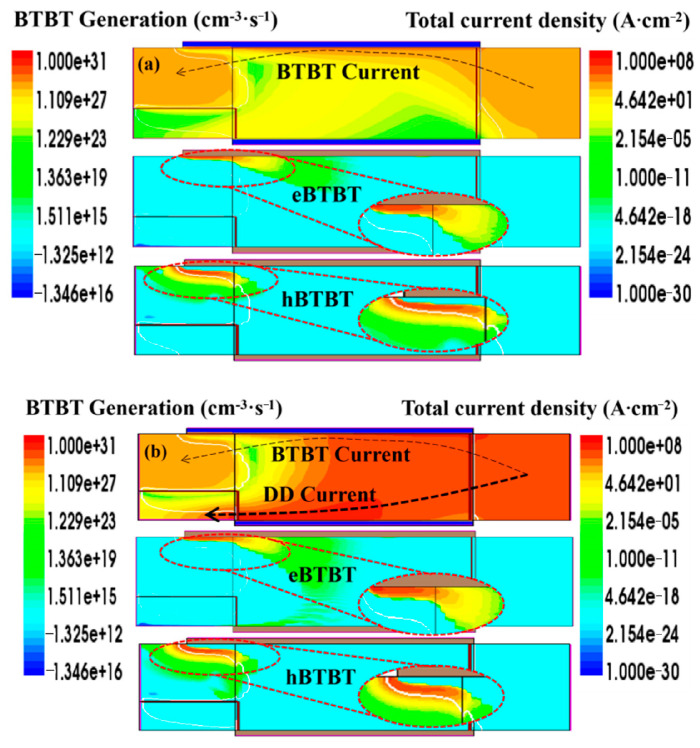
The BTBT rate and current density at (**a**) V_GS_ = 0.3 V and (**b**) V_GS_ = 0.9 V.

Figure 3a presents the distribution of the current density and carrier tunneling probability for V_GS_ = 0.3 V. It can be seen that the current is mainly concentrated in the channel region near the top gate and that the carrier tunneling probability is mainly distributed in the P+ source region. Moreover, the electron tunneling probability (eBTBT) is closely distributed near the interface between the P+ source and the top gate, while the distribution of the hole tunneling probability (hBTBT) is farther away from this interface, indicating that the tunneling process is perpendicular to the interface between the P+ source and the top gate. Specifically, the electrons tunnel from the region in the P+ source farther away from the top gate interface to the region close to the top gate interface. Subsequently, the tunneling electrons drift towards the drain direction under the effect of the drain voltage until they are collected by the drain contact and form the drain current. Therefore, at V_GS_ = 0.3 V, the BTBT dominates the device current being distributed in the channel near the top gate interface.

When V_GS_ gradually increases to 0.9 V, it is large enough to induce a large number of electrons in the channel region near the bottom surface, thus connecting the N+ source to the N+ drain so that the electrons in the N+ source can enter the channel region near the bottom gate by thermal emission. These electrons then continue to drift in the channel and are finally collected by the drain electrode. Considering that the probability of carrier thermal emission is much higher than the probability of carrier tunneling, the drift current is significantly higher than the BTBT current mainly concentrated near the top gate interface. Therefore, the drift-diffusion mechanism dominates the device current, and this is why the device current is mainly distributed in the channel near the bottom surface, although the tunneling probability and tunneling current are greatly enhanced, as exhibited in Figure 3b.

**Figure 4:**
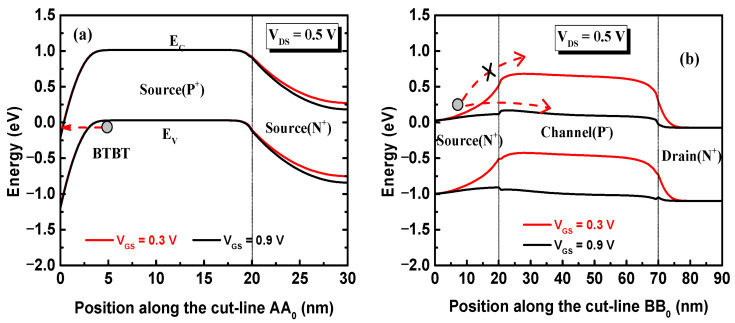
The energy band diagrams along the cut-lines (**a**) AA_0_ and (**b**) BB_0_.

In order to further illustrate the conduction mechanism of the TDDFET, the band diagrams along the cut-line AA_0_ are given in Figure 4a. Whether V_GS_ = 0.3 V or 0.9 V, the conduction band in the P+ source region close to the interface between the top gate and the P+ source region is lower than the valence band in the region farther away from the interface. This allows the electrons in the region farther away from the interface between the P+ source and the top gate to tunnel into the region closer to the interface. Finally, the electron tunneling probabilities are distributed in the region close to the top gate in the P+ source region, and the hole tunneling probabilities are distributed in the region far away. The band diagram along the cut-line BB_0_ is presented in Figure 4b. Obviously, at a low voltage V_GS_ = 0.3 V, there is a high energy barrier near the interface between the N+ source region and the channel, preventing the electrons in the N+ source from injecting into the channel, resulting in a low drift-diffusion current. Therefore, the device current at V_GS_ = 0.3 V is dominated by the tunneling mechanism and is mainly distributed in the channel body near the top surface. However, at V_GS_ = 0.9 V, the interface barrier is greatly reduced, and the electrons in the N+ source can be injected into the channel region through thermal emission and gradually drift towards the drain direction until they are collected by the drain electrode. Because the probability of thermal emission is much higher than that of tunneling, the device current is dominated by the drift-diffusion mechanism and is mainly distributed in the channel body near the bottom surface.

In TDDFETs, two conduction mechanisms are at play. When V_GS_ < V_turn_, the device current mainly depends on the tunneling mechanism and is distributed in the channel body near the top surface. When V_GS_ > V_turn_, the device current mainly depends on the drift-diffusion mechanism and is distributed in the channel body near the bottom surface. Since the drift-diffusion current is significantly larger than the tunneling current, the current curve turns around V_turn_ and makes the TDDFET present the third state between the on state and the off state. Thus, the TDDFET is very suitable for the design of ternary logic circuits.

## 3. Ternary Logic Circuit Design

This part introduces the design of some ordinary ternary logic circuits using the TDDFETs, including the ternary combinational and sequential logic circuits. Owing to the absence of analytical models for TDDFETs and the time-consuming and challenging convergence nature of numerical simulations for multi-device circuits, the widely used lookup table approach is adopted. In this approach, the TDDFET is treated as a black box, and the corresponding electrical characteristics are simulated through interpolation from a table containing a large amount of input–output data of the device. Thus, a greater volume of input–output data results in enhanced model accuracy. In this part, the TCAD numerical simulation is used to generate the table containing a large number of Current–Voltage (I–V) and Capacitance–Voltage (C–V) data of the n- and p-type TDDFETs, and then, these data are embedded into the SPICE simulation tool using the Verilog-A language. To ensure the accuracy of the simulation, the current and capacitance of the TDDFET change with the variation in V_DS_ sweeping V_GS_ from 0.0 V to 1.5 V in small increments of 0.03 V.

The inverter is the most basic unit circuit in the ternary logic circuits, and there are three kinds of ternary inverters, namely, the Standard Ternary Inverter (STI), Negative Ternary Inverter (NTI), and Positive Ternary Inverter (PTI). The truth tables are shown in Table 2, and the difference between these three inverters is that the output corresponding to the input logic “1” is different. The STI, NTI, and PTI invert input logic “1” to logic “1”, “0”, and “2”, respectively.

**Figure 5:**
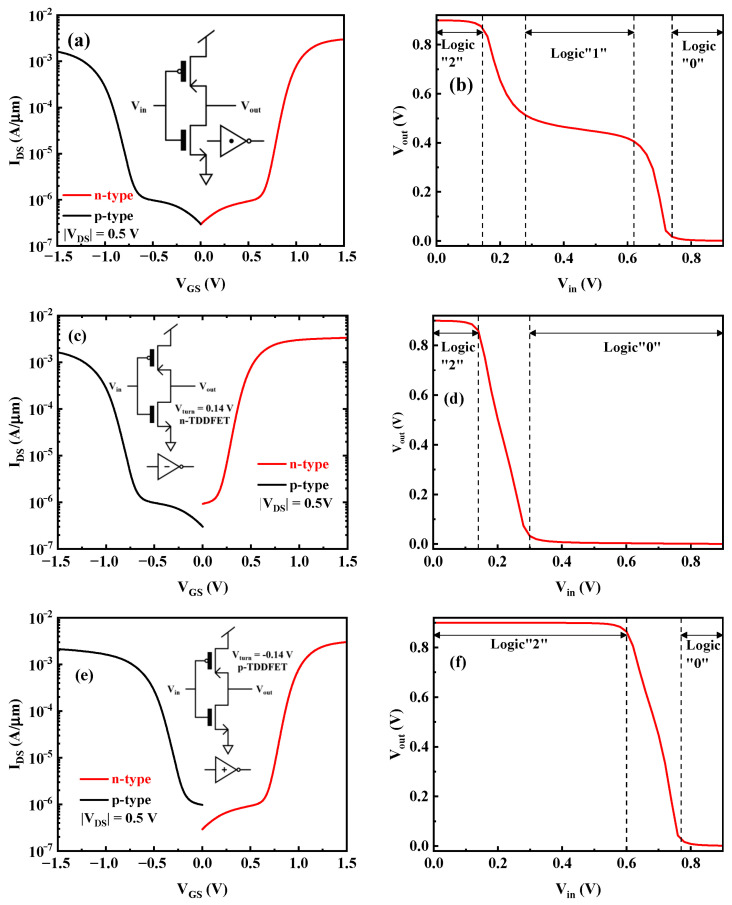
The transfer curves of the TDDFETs and VTCs for the (**a**,**b**) STI, (**c**,**d**) NTI, and (**e**,**f**) PTI, respectively.

Figure 5a shows the almost-symmetrical n-type and p-type TDDFET transfer curves, and the corresponding voltage transfer curve (VTC) of the STI at V_DD_ = 0.9 V is presented in Figure 5b. The circuit structure of the STI built by the TDDFET shown in the insert in Figure 5a is exactly the same as that of the binary inverter. When the input logic is “0”, the input voltage V_in_ is very small and close to 0.0 V, the gate-source voltage of the nTDDFET, V_GSn_ = V_in_, is also small, and the nTDDFET is in the off state. The gate-source voltage of the p-type TDDFET (pTDDFET) V_GSp_ = |V_in_ − V_DD_| is large, and the pTDDFET is in the on state. Finally, the output V_out_ ≈ V_DD_ according to the series voltage division and the output logic “2” are obtained. When the input logic is “2”, the input voltage V_in_ is large and close to V_DD_. Thus, V_GSn_ = V_in_ is large, and the nTDDFET is in the on state. V_GSp_ = |V_in_ − V_DD_| is small, and the pTDDFET is in the off state. Therefore, V_out_ ≈ 0.0 V, and the output logic “0” is obtained. When the input Vin is near V_DD_/2 = 0.45 V, that is, when the input logic is “1”, V_GSn_ = V_GSp_ = 0.45 V. Thus, the nTDDFET and pTDDFET are both in the BTBT region, as exhibited by the transfer curves. Owing to the almost-symmetrical transfer curves, the BTBT current and output resistance of the two devices in the BTBT region are similar; thus, based on the series voltage division, it can be obtained that V_out_ ≈ V_DD_/2 = 0.45 V. That is, the output logic “1” is obtained.

Different from the STI, the NTI inverts input logic “1” to logic “0”, as its name indicates. This requires the current (or resistance) of the nTDDFET to be significantly greater than (or less than) the current (or resistance) of the pTDDFET when the input V_in_ is near V_DD_/2. This can be achieved by shifting the nTDDFET transfer curve to the left, which can be obtained by appropriately reducing the WF_1_ and WF_2_. Figure 5c shows the transfer curves of the nTDDFET with reduced V_turn_ = 0.14 V and that of the pTDDFET with unchanged V_turn_. It can be seen that when V_in_ is near V_DD_/2 = 0.45 V, that is, when the input logic is “1”, V_GSp_ = |V_in_ − V_DD_| = 0.45 V, which causes the pTDDFET to be in the BTBT region. However, when V_GSn_ = V_in_ = 0.45 V, the nTDDFET is in the DD region owing to the reduced V_turn_. The output resistance of the nTDDFET in the DD region is much smaller than that of the pTDDFET in the BTBT region. Thus, V_out_ ≈ 0.0 V, and the output logic “0” is obtained, as shown by the VTC curve of the NTI presented in Figure 5d.

Similarly, if the PTI is to be realized, namely, the input logic “1” is reversed to logic “2”, we need to shift the transfer curve of the pTDDFT to the right by appropriately increasing the WF_1_ and WF_2_. Figure 5e shows the transfer curves of the pTDDFET with right-shifted V_turn_ = −0.14 V and the nTDDFET with unchanged V_turn_. When the input V_in_ is near V_DD_/2 = 0.45 V, that is, the input logic is “1”. When V_GSn_ = V_in_ = 0.45 V, the nTDDFET is in the BTBT region, while when V_GSp_ = |V_in_ − V_DD_| = 0.45 V, the pTDDFET is in the DD region. The output resistance of the pTDDFET in the DD region is much smaller than that of the nTDDFET in the BTBT region. Therefore, V_out_ ≈ V_DD_ = 0.9 V, and the output logic “2” is obtained, as shown by the VTC of the PTI exhibited in Figure 5f.

**Figure 6:**
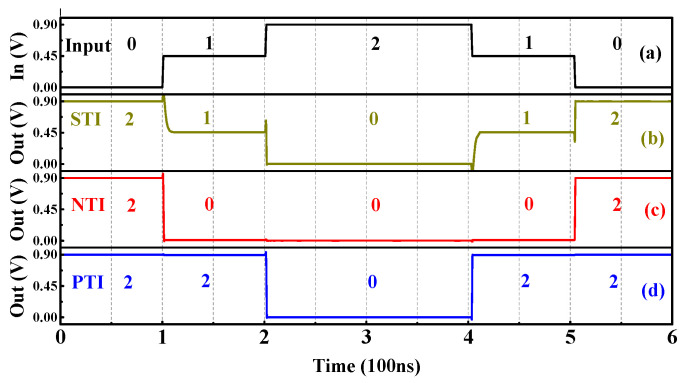
The (**a**) input voltage and corresponding output voltage for the (**b**) STI, (**c**) NTI, and (**d**) PTI.

The three types of ternary inverters can be realized by properly designing the V_turn_ of the TDDFETs. Considering that the circuit structure of the ternary inverter is the same as that of the binary inverter, the circuit symbols of the STI, NTI, and PTI are similar to the binary inverter, as shown in Figure 5a,c,e. The difference is that “·”, “−“, and “+”, respectively, refer to the STI, NTI, and PTI. Figure 6 shows the transient performance of the three inverters, and the expected function is obtained.

**Figure 7:**
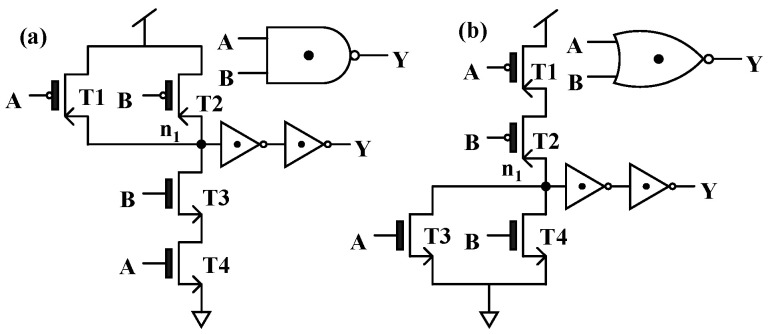
The circuits and symbols of the (**a**) T-NAND and (**b**) T-NOR.

**Figure 8:**
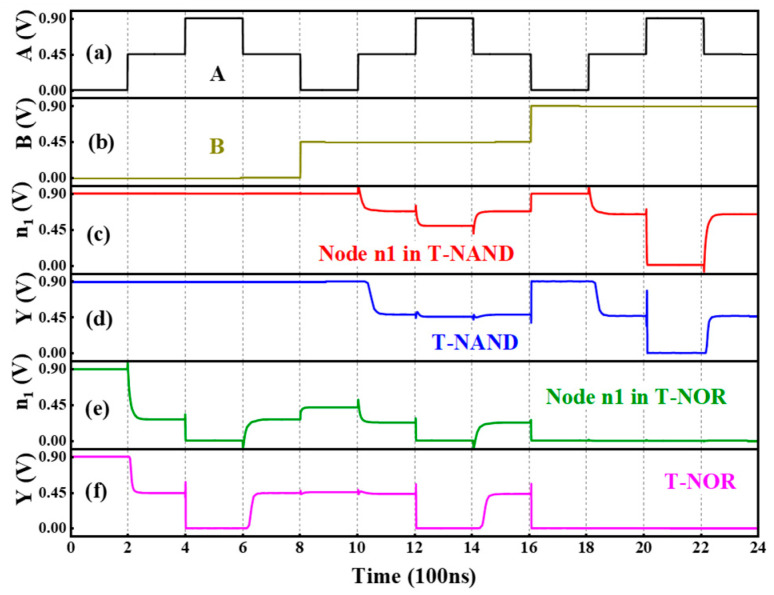
The inputs (**a**) A and (**b**) B, (**c**) the voltage at node n1, (**d**) the output for the T-NAND, (**e**) the voltage at node n1, and (**f**) the output for the T-NOR.

Table 3 gives the truth tables of the T-NAND and T-NOR, and the corresponding circuits and symbols are shown in Figure 7. It should be noted that when the TDDFET is in the off or on state, it can be approximately equivalent to an open or short circuit, and the voltage at node n1 can be approximately equal to 0.0 V or V_DD_. This is the same as that in a binary inverter. Taking the T-NAND in Figure 7a as an example, for input logic B = “0”, T2 is in the on state and can be approximately considered as a short circuit, whereas T3 is in the off state and can thus be approximately considered as an open circuit. Therefore, regardless of the input value of A, the equivalent resistance of the pull-up path is approximately 0 Ω, while the equivalent resistance of the pull-down path is approximately infinite; so, the voltage at node n1 is approximately V_DD_ and the output logic is kept as “2”, as presented in Figure 8c, which gives the transient performance of the T-NAND. To achieve logic “0” at node n1, the equivalent resistance of the pull-down path should be close to 0 Ω, while that of the pull-up path should be extremely high. This implies that T1 and T2 can operate as open circuits, while T3 and T4 act as short circuits, which requires that both the inputs A and B should be logic “2”, as presented in Figure 8c.

To obtain the logic “1” at node n1, the equivalent resistance of the pull-up path must be equal to that of the pull-down path. Many combinations of the inputs A and B can lead to a voltage of V_DD_/2 at node n1. However, it should be noted that the TDDFET cannot be simply approximated as an open circuit or a short circuit when it operates in the BTBT region. This would result in unequal equivalent resistance of the pull-up and pull-down paths and further lead to the voltage at node n1 deviating from the expected value V_DD_/2. Assuming that the resistance of the nTDDFET and pTDDFET in the BTBT region is equal and that both are *R*, when the input of the T-NAND is A = B = “1”, T1–T4 are all in the BTBT region; then, the equivalent resistance of the pull-up path is *R*/2, while that of the pull-down path is 2*R*. As a result, the voltage at the node n1 is 4 V_DD_/5, relatively higher than the expected output V_DD_/2, as shown in Figure 8c. This deviation may be amplified step by step in subsequent circuits and result in an error logic output. In order to address this problem, two STIs are connected to node n1. In this way, when Y outputs logic “1”, the equivalent resistance of the pull-up and pull-down paths is always equal, and, thus, the output voltage can be kept near V_DD_/2 for the output logic “1”, as shown in Figure 8d.

The analysis of the T-NOR is similar to that of the T-NAND, except that when A = B= “1”, that is, T1–T4 are all in the BTBT region, the equivalent resistance of the pull-up path is 2*R*, while that of the pull-down path is *R*/2. The opposite is true for the T-NAND, leading to the voltage at node n1 being V_DD_/5, lower than the expected V_DD_/2, as shown in Figure 8e. Again, this problem can be solved by connecting two STIs, as shown in Figure 8f. It should be noted that, although the two STIs enhance the anti-interference ability, they also sacrifice the circuit speed and energy consumption. Actually, two STIs can achieve the function of the T-NAND and T-NOR. For the ternary circuits based on the T-AND and T-NOR, only one STI is enough.

**Figure 9:**
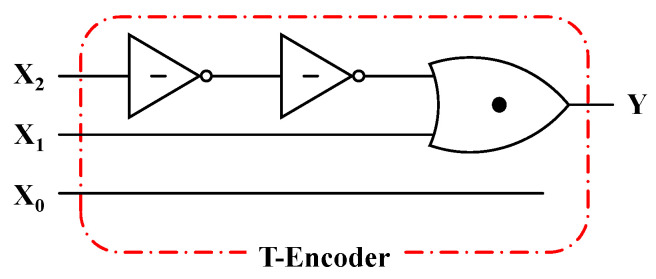
The circuit of the T-Encoder.

**Figure 10:**
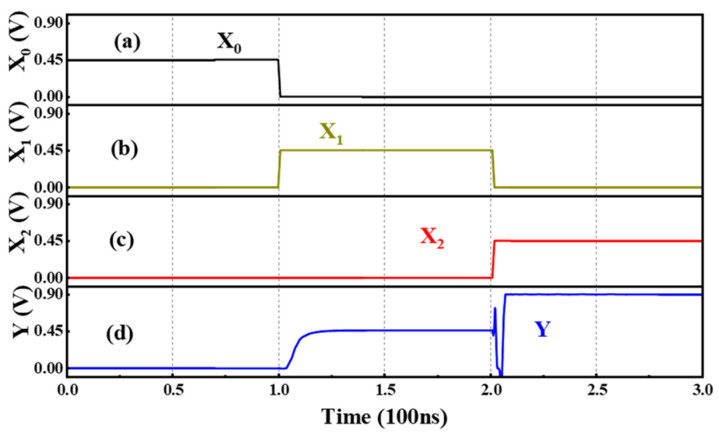
The inputs (**a**) X_0_, (**b**) X_1_, (**c**) X_2_ and output (**d**) Y of the T-Encoder.

In addition to the basic units, the Ternary Encoder (T-Encoder) with three inputs and one output is also designed, and the truth table is given in Table 4. Obviously, the encoder encodes three different states, namely, “0”, “1”, and “2”, respectively. Only one of the three inputs is allowed to be valid at a time; so, it is not a priority encoder. The logic “1” is used to represent the effective input signal; then, the output Y can be expressed as Y = ((X_2_)_N_′)_N_′ + X_1_, indicating that the input signal X_2_ is added to X_1_ after two NTI inverters. The circuit diagram is shown in Figure 9 and Figure 10 shows the transient performance. It can be seen that the circuit can achieve the expected function of the Ternary Encoder.

**Figure 11:**
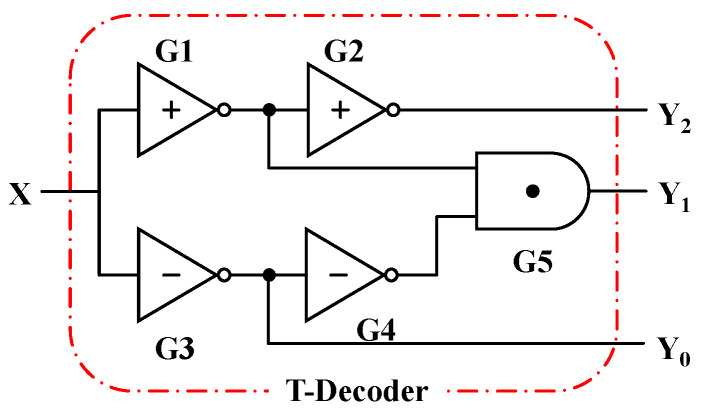
The circuit of the T-Decoder.

**Figure 12:**
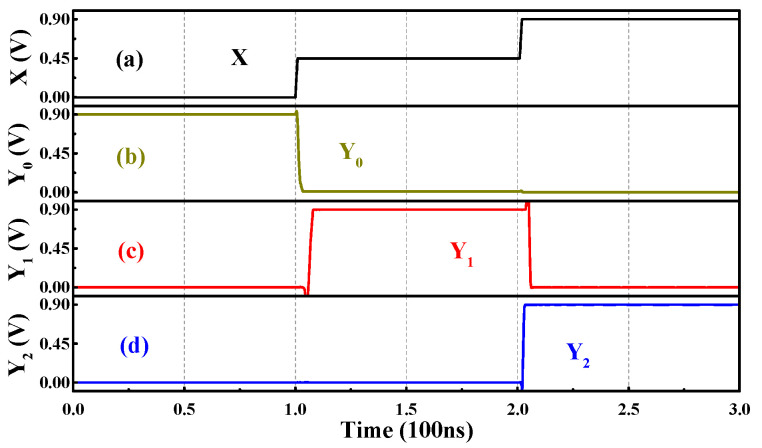
The input (**a**) X and outputs (**b**) Y_0_, (**c**) Y_1_, (**d**) Y_2_ of the T-Decoder.

The decoder is the inverse operation of the encoder and the truth table of the Ternary Decoder (T-Decoder) with one input and three outputs, as presented in Table 5. The input signal X can be “0”, “1”, and “2”, corresponding to three different output ports, namely, Y_0_, Y_1_, and Y_2_, respectively. When the input signal selects a certain port, this port outputs the valid signal “2”, while the other ports output “0”; only one of the three outputs can be valid at a time. The circuit structure of the Ternary Encoder is given in Figure 11, and Figure 12 presents the transient curves.

**Figure 13:**
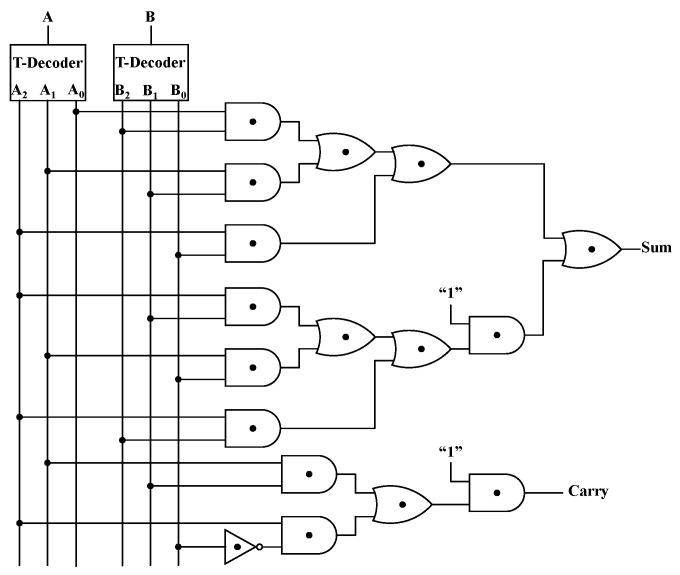
The circuit diagram of the T-HA.

**Figure 14:**
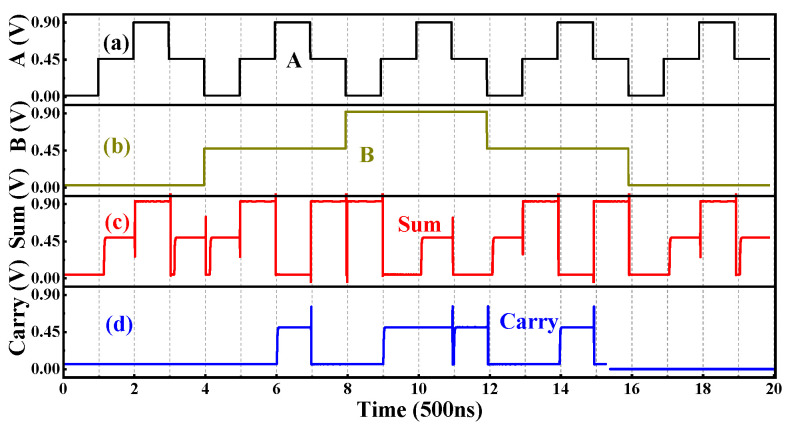
The inputs (**a**) A, (**b**) B and outputs (**c**) sum (**d**) carry of the T-HA.

A Ternary Half Adder (T-HA) adds two ternary numbers A and B to produce a Sum and a Carry, without considering the Carry from the lower adder. The truth table is presented in Table 6, in which {A_0_, A_1_, A_2_} and {B_0_, B_1_, B_2_} are the outputs of the decoder with inputs A and B, respectively. When the Sum is less than “3”, the Carry is “0”. When the Sum is greater than or equal to “3”, it needs to be carried to a higher adder, and, thus, the Carry is “1”. The Sum and Carry can be expressed as Sum = A_0_B_2_ + A_1_B_1_ + A_2_B_0_ + 1 (A_0_B_1_ + A_1_B_0_ + A_2_B_2_) and Carry = 1 (A_1_B_2_ + A_2_B_0′_), respectively. Figure 13 gives the circuit structure of the T-HA, and the outputs for the inputs are exhibited in Figure 14, which shows that the T-HA can work properly.

**Figure 15:**
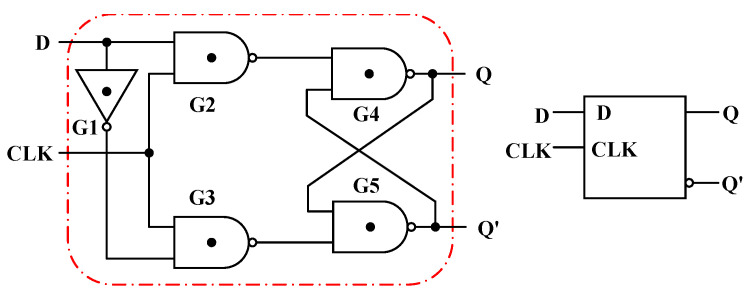
The circuit and symbol of the Ternary D-Latch.

**Figure 16:**
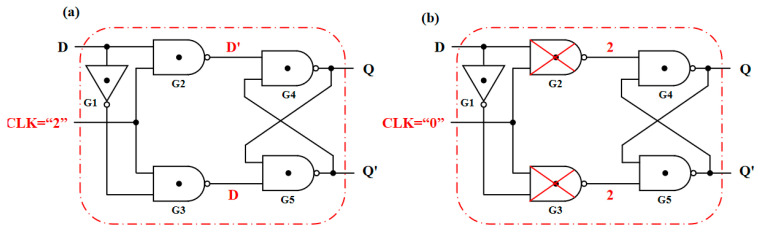
The Ternary D-Latch with (**a**) CLK = “2” and (**b**) CLK = “0”.

**Figure 17:**
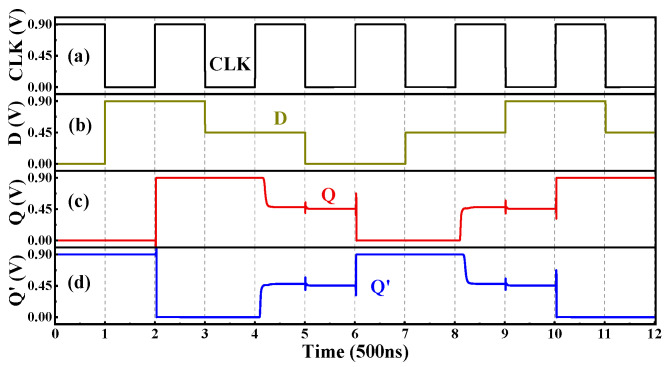
The (**a**) CLK, (**b**) input D, (**c**) output Q, and (**d**) Q′ of the Ternary D-Latch.

In addition to the combinational logic circuits, the sequential logic D-Latch based on the T-NAND is also studied and shown in Figure 15. When the clock signal CLK = “2”, it can be seen from the truth table of the T-NAND that the gates G2 and G3 output D’ and D, respectively, as shown in Figure 16a. In addition, the output of gate G4 is connected to the input of gate G5, and the output of gate G5 is connected to the input of gate G4, forming a feedback loop. Table 7 gives the state transition of the gates G4 and G5 with the inputs D’ and D, in which the Q_n_ and Q_n+1_ are the current state and the next state, respectively. The state output equation is Q_n+1_ = D. When CLK = “0”, the outputs of the gates G2 and G3 are kept at logic “2”; that is, the gates G2 and G3 are locked, and the input signal D cannot pass through gates G2 and G3, as shown in Figure 16b. At this time, the gates G4 and G5 with an input logic of “2” are equivalent to two STIs in a positive feedback connection. Thus, the stored internal signals can remain unchanged, namely, the state output equation is Q_n+1_ = Q_n_. In summary, when CLK = “0”, the output remains unchanged, and when CLK = “2”, the input signal can be transferred to the output. Thus, this D-Latch is lever-triggered, and the transient curves are shown in Figure 17.

**Figure 18:**
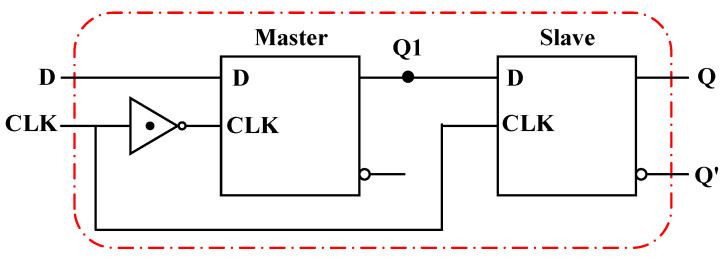
The circuit of the ternary edge-triggered D-FF.

**Figure 19:**
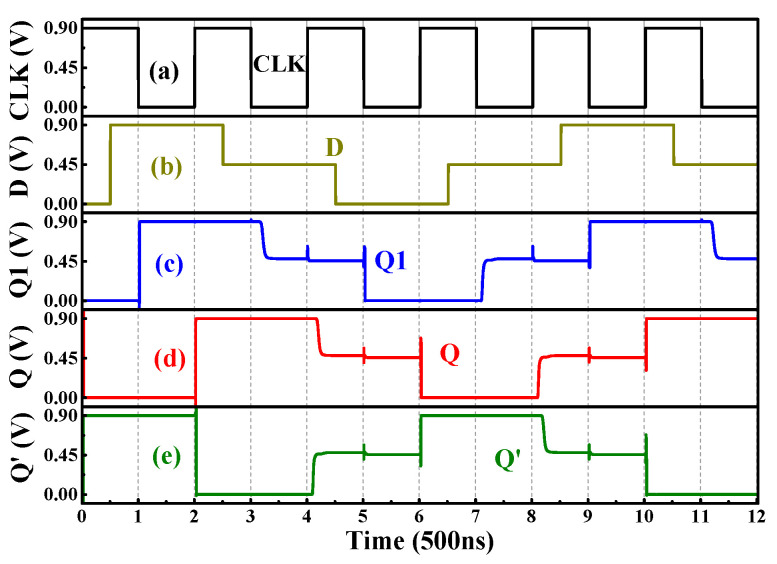
The (**a**) clock signal, (**d**) input D, (**c**) signal at node Q1, (**d**) output Q and (**e**) output Q′ of the ternary edge-triggered D-FF.

Two Ternary D-Latches can be cascaded to form the master–slave structure to obtain the ternary edge-triggered D-FF, as shown in Figure 18. When CLK = “0”, the slave FF is in the hold state and does not receive any data, while the output Q1 of the master FF varies with the input signal D. The input data D at the last moment during CLK = “0” are stored at the output node Q1 of the master FF. When CLK = “2”, the master FF is in the hold state, the logic at node Q1 does not vary, and the slave FF transmits the data stored at Q1 to the output Q. In this way, the input data at the last moment during CLK = “0” are transferred to the output Q at the rising edge of the CLK, as presented in Figure 19, exhibiting the transient curves of the circuit.

## 4. Conclusions

TDDFETs are proposed for a ternary logic design based on the combination of the tunneling and drift-diffusion mechanisms. The TDDFET presents three stable states and is suitable for the design of ternary logic circuits. In this paper, the principle of the TDDFET is analyzed in detail and is based on the table lookup method; the TDDFET is packaged as a “black box” in the SPICE platform. Using the Verilog-A language, the basic unit circuits, such as STI, NTI, PTI, T-NAND, and T-NOR, are designed based on the proposed TDDFETs. In order to solve the problem of voltage deviation in the T-NAND and T-NOR, two STIs are cascaded at the output to ensure stability and reliability. Moreover, based on the unit circuits, the Ternary Encoder, Ternary Decoder, Ternary Half Adder, Ternary D-Latch, and edge-triggered D-FF are built, indicating the important significance of the proposed TDDFETs for the future investigation of the ternary logic circuits.

## Figures and Tables

**Table 1:** The parameters of the n-type TDDFET.

Symbols	Parameters	Values
*T_sn_*	Thickness of the N^+^ source	10 nm
*T_sp_*	Thickness of the P^+^ source	20 nm
*T_ox_*	Thickness of the gate oxide	2 nm
*L_ch_*	Length of the channel	50 nm
*L_ov_*	Overlap between the top gate and the source	10 nm
*N_sn_*	Doping density of the N^+^ source	1 × 10^20^ cm^−3^
*N_sp_*	Doping density of the P^+^ source	1 × 10^20^ cm^−3^
*N_ch_*	Doping density of the channel	1 × 10^16^ cm^−3^
*N_d_*	Doping density of the drain	1 × 10^19^ cm^−3^
*WF*_1_	Work function of the top gate	3.10 eV
*WF*_2_	Work function of the bottom gate	4.92 eV

**Table 2:** The truth tables of the STI, NTI, and PTI.

In	Out		
	STI	NTI	PTI
0	2	2	2
1	1	0	2
2	0	0	0

**Table 3:** The truth tables of the T-NAND and T-NOR.

**T-NAND**		**B**			**T-NOR**		**B**		
		0	1	2			0	1	2
**A**	0	2	2	2	**A**	0	2	1	0
	1	2	1	1		1	1	1	0
	2	2	1	0		2	0	0	0

**Table 4:** The truth table of the T-Encoder.

X_0_	X_1_	X_2_	Y
1	0	0	0
0	1	0	1
0	0	1	2

**Table 5:** The truth table of the T-Decoder.

X	Y_2_	Y_1_	Y_0_
0	0	0	2
1	0	2	0
2	2	0	0

**Table 6:** The truth table of the T-HA.

A	B	A_0_	A_1_	A_2_	B_0_	B_1_	B_2_	Sum	Carry
0	0	2	0	0	2	0	0	0	0
0	1	2	0	0	0	2	0	1	0
0	2	2	0	0	0	0	2	2	0
1	0	0	2	0	2	0	0	1	0
1	1	0	2	0	0	2	0	2	0
1	2	0	2	0	0	0	2	0	1
2	0	0	0	2	2	0	0	2	0
2	1	0	0	2	0	2	0	0	1
2	2	0	0	2	0	0	2	1	1

**Table 7:** The truth table of the Ternary D-Latch.

D	D′	Qn	Qn′	Qn + 1	Qn + 1′
0	2	0	2	0	2
1	1	0	2	1	1
2	0	0	2	2	0
0	2	1	1	0	2
1	1	1	1	1	1
2	0	1	1	2	0
0	2	2	0	0	2
1	1	2	0	1	1
2	0	2	0	2	0

## Data Availability

The original contributions presented in this study are included in the article. Further inquiries can be directed to the corresponding author.
